# GPs’ mindlines on deprescribing antihypertensives in older patients with multimorbidity: a qualitative study in English general practice

**DOI:** 10.3399/bjgp21X714305

**Published:** 2021-05-18

**Authors:** Karolina Kuberska, Fiona Scheibl, Carol Sinnott, James P Sheppard, Mark Lown, Marney Williams, Rupert A Payne, Jonathan Mant, Richard J McManus, Jenni Burt

**Affiliations:** National Institute for Health Research senior clinical lecturer in general practice;; Norwich Medical School, University of East Anglia, Norwich.; National Institute for Health Research senior clinical lecturer in general practice;; Nuffield Department of Primary Care Health Sciences, University of Oxford, Oxford.; Primary Care Research Group, University of Southampton, Southampton.; Patient and public involvement representative, London.; Centre for Academic Primary Care, University of Bristol, Bristol.; Department of Public Health and Primary Care, University of Cambridge, Cambridge.; Nuffield Department of Primary Care Health Sciences, University of Oxford, Oxford.; The Healthcare Improvement Studies Institute (THIS), University of Cambridge, Cambridge.

**Keywords:** general practice, polypharmacy, hypertension, deprescriptions, aged, 80 and over, qualitative research

## Abstract

**Background:**

Optimal management of hypertension in older patients with multimorbidity is a cornerstone of primary care practice. Despite emphasis on personalised approaches to treatment in older patients, there is little guidance on how to achieve medication reduction when GPs are concerned that possible risks outweigh potential benefits of treatment. Mindlines — tacit, internalised guidelines developed over time from multiple sources — may be of particular importance in such situations.

**Aim:**

To explore GPs’ decision-making on deprescribing antihypertensives in patients with multimorbidity aged ≥80 years, drawing on the concept of mindlines.

**Design and setting:**

Qualitative interview study set in English general practice.

**Method:**

Thematic analysis of face-to-face interviews with a sample of 15 GPs from seven practices in the East of England, using a chart-stimulated recall approach to explore approaches to treatment for older patients with multimorbidity with hypertension.

**Results:**

GPs are typically confident making decisions to deprescribe antihypertensive medication in older patients with multimorbidity when prompted by a trigger, such as a fall or adverse drug event. GPs are less confident to attempt deprescribing in response to generalised concerns about polypharmacy, and work hard to make sense of multiple sources (including available evidence, shared experiential knowledge, and non-clinical factors) to guide decision-making.

**Conclusion:**

In the absence of a clear evidence base on when and how to attempt medication reduction in response to concerns about polypharmacy, GPs develop ‘mindlines’ over time through practicebased experience. These tacit approaches to making complex decisions are critical to developing confidence to attempt deprescribing and may be strengthened through reflective practice.

## INTRODUCTION

There is robust evidence for the benefits of prescribing antihypertensive medication,^1^^–^^4^ including for the oldest patients.^5^ Consequently, official guidelines focus on the need to prescribe,^6^^,^^7^ and the Quality and Outcomes Framework (QOF; the English general practice payment-for-performance system) sets GPs clear targets for achieving controlled blood pressure in patients under their care.^8^ More recent iterations of the QOF have, however, recognised the importance of personalised treatment approaches for frail older patients with multimorbidity.^8^ GPs are encouraged to use clinical judgement when choosing to exempt patients from QOF indicator targets (measures against which practice performance is assessed); this may include decisions to reduce rather than continue medication, including antihypertensives.^9^^–^^15^

How, though, does such clinical judgement develop, and what sources do GPs draw on in reaching prescribing decisions, especially those that relate to stopping or reducing medicines? The processes involved in clinical decision-making have been the focus of much research, which highlights the gap between the rational, information-driven approach of evidence-based guidelines and the messy realities of clinical practice on the ground.^16^^–^^19^ Gabbay and le May describe the development of clinical decision-making in GPs using the concept of ‘mindlines’:

*‘… collectively reinforced, internalised tacit guidelines, which were informed by brief reading, but mainly by their* [doctors’] *interactions with each other and with opinion leaders, patients, and pharmaceutical representatives and by other sources of largely tacit knowledge that built on their early training and their own and their colleagues’ experience.’* ^20^

Thus, GPs’ decision-making, informed in part by available evidence and guidance, is refined through reference to the outcomes of previous decisions, whether their own or their colleagues’. Information is negotiated, shared, and tested in a community of practice on an ongoing basis, with iterative development of in-practice knowledge.^20^^,^^21^

Importantly, mindlines develop and strengthen continually in response to newly emerging evidence, as well as the need to act.^22^ GPs are faced with decisions around treatment for frail, older, hypertensive patients every day. Here, they are required to make trade-offs between evidence-supported benefits of antihypertensive treatment and potential benefits of deprescribing. Relevant ‘brief reading’ includes recommendations on how to recognise and action opportunities for deprescribing,^23^^–^^25^ and National Institute for Health and Care Excellence (NICE) guidance on multimorbidity, none of which offers advice on reducing specific groups of medication, including antihypertensives.^26^ These reflect the lack of current evidence about the advantages of withdrawing antihypertensive medication.^5^^,^^12^^,^^13^^,^^27^

**Table:** How this fits in

While there is robust evidence for the benefits of prescribing antihypertensive medication in healthy older patients, the balance of probable benefit against potential risk is less certain in older patients with multimorbidity. An emphasis on the importance of clinical judgement in prescribing decisions for such patients, seen for example in recent revisions to the Quality and Outcomes Framework (QOF) to support person-centred treatment goals, is not yet highlighted in specific guidelines on how to attempt medication reduction. In this examination of how GPs develop and apply their clinical judgement in relation to medication reduction in older patients with multimorbidity, decisions to deprescribe were typically based on clear trigger events or direct requests from patients. GPs found it far harder to come to a decision to deprescribe in response to a generalised concern about polypharmacy: here, experiential knowledge, accrued over time and through multiple sources (mindlines), was critical to developing confidence in deprescribing in the absence of robust medication reduction guidelines.

With little information on the safety and efficacy of medication reduction, increased interest in deprescribing for older patients^16^^,^^28^^–^^34^ has had minimal impact on clinical practice.^30^^,^^32^^,^^35^^,^^36^ To date, studies have focused on barriers and facilitators to deprescribing preventive medication.^13^^,^^15^^,^^16^^,^^29^^,^^30^^,^^34^^,^^36^^–^^41^ Questions that have received far less attention include: what shapes the decision-making of GPs at the sharp end in managing hypertension in frail older patients; how frameworks of knowledge are, individually and collectively, being created and maintained in response to increased attention towards polypharmacy concerns; and how GPs might learn from these in developing their own decision-making in this area.

This qualitative study took place as part of the OPtimising Treatment for MIld Systolic hypertension in the Elderly trial (OPTiMISE), whose goal was to compare a strategy of antihypertensive medication reduction with usual care for older (aged ≥80 years) patients with multimorbidity in primary care.^42^ The aim was to explore how GPs made decisions about prescribing and deprescribing antihypertensives in older patients with multimorbidity, within everyday clinical practice.

## METHOD

### Design

This study was a qualitative study using chart-stimulated recall approaches to explore GPs’ clinical decision-making on treatment for older patients with hypertension, set in English general practice. With the support of a Clinical Research Network, the authors aimed, as far as possible, to recruit a mixed sample of GPs across the following two criteria: length of time qualified (<10 or ≥10 years); and practice location (rural, urban, or mixed).

### Data collection

The authors conducted semi-structured face-to-face interviews with GPs, lasting between 20–40 minutes. A chart-stimulated recall method was used, which is a case-based technique used to examine clinical decision-making, taking account of contextual influences on approaches to patient care.^17^ It has been used in a number of GP interview studies, including those exploring management of multimorbidity and hypertension.^18^ Interviews followed a two-phase approach. GPs were asked to reflect on two cases, whom they selected from their current patient list according to the following criteria: aged ≥80 years; controlled blood pressure (systolic blood pressure <150 mmHg); and receiving ≥2 antihypertensive medications and whom the GP considers may benefit from medication reduction due to existing polypharmacy, comorbidity, and frailty.

For each patient, GPs were asked to consider their approach to the management of their patients’ hypertension; how this was balanced with their other medical conditions; what had influenced their management approach for this case; whether additional knowledge would have further informed their decision-making; and how they would think about stopping an antihypertensive medication. The interviews concluded with a second, more open phase of questioning, asking GPs to explain their approach to the management of hypertension in older patients, and the circumstances and outcomes of any previous decisions to stop antihypertensive medication. Sample sufficiency was guided by the principles of information power.^43^

All interviews were conducted by a single, non-clinical interviewer, audiorecorded, and professionally transcribed. A pilot interview took place with one GP to assess the feasibility of the topic guide.

### Analysis

This study used a thematic analysis approach,^44^ with analysis commencing alongside data collection. As interviews were conducted, two researchers who were trained in qualitative methods read the transcripts and iteratively developed first- and second-level coding frameworks as analysis progressed.^45^ As thinking moved from descriptive to analytical, the team drew on existing literature on GPs’ attitudes to medication reduction and deprescribing, as well as models of understanding clinical decision-making in conditions of uncertainty, notably the concept of mindlines.^20^^,^^21^^,^^46^^,^^47^ Working with transcripts, four researchers trained in qualitative methods and general practice subsequently discussed data and thematic development in three half-day workshops. A revised coding framework was developed following each workshop. As a part of data analysis, the major themes identified were discussed at a multidisciplinary daylong analysis workshop attended by GPs, cardiovascular physicians, social scientists, and a patient representative. This workshop was repeated on completion of data collection and analysis, with final reflection on, and discussion of, the core findings, including the application and relevance of the concept of mindlines to the data. For example, in developing second-level coding frameworks, the team reflected on how and where key concepts were apparent in the data (such as how multiple sources of knowledge appeared to be distilled and employed by GPs in managing hypertension). Thus, these constructs were used as a tool to think about the data, rather than in a deductive approach to framework development and coding.

## RESULTS

This study interviewed 15 GPs from seven practices based in the East of England between May 2017 and October 2017. Three practices were rural, three were mixed, and one was urban. List size ranged from 4000 to 11 500. Four GPs were female and 11 were male; professional experience ranged from 1 month to 26 years (Table 1).

**Table 1.** Summary details of practices and participants in GP interview study

**Practice ID**	**Area**	**Patient list size, *n***	**GPs interviewed, *n***	**Age range, years**	**Sex**	**Range of years at practice, years**
**Practice 1**	rural	8000	4	30–35	male	<5
				45–50	male	5–10
				45–50	male	10–15
				50–55	male	20–25

**Practice 2**	mixed	9000	1	50–55	male	5–10

**Practice 3**	mixed	9500	2	50–55	female	25–30
				55–60	male	10–15

**Practice 4**	urban	11 500	2	30–35	male	<5
				35–40	female	5–10

**Practice 5**	mixed	9500	1	40–45	male	5–10

**Practice 6**	rural	7000	3	30–35	male	<5
				45–50	male	10–15
				45–50	female	10–15

**Practice 7**	rural	4000	2	55–60	male	<5
				55–60	female	5–10

Interviews with GPs illustrated the complexity of everyday decisions to deprescribe antihypertensive medication in patients with multimorbidity who are ≥80 years old in the absence of specific guidance. Although clinical considerations (such as presence of adverse drug effects, recent falls, or drug interactions) were at the forefront of GPs’ concerns, their impact on medication reduction decisions was not always straightforward. GPs consistently highlighted the lack of explicit mechanisms for medication reduction, fuelling the importance of tacit, experiential, and non-clinical considerations in decisions concerning medication regimens of older patients with multimorbidity.

**Figure 1.**
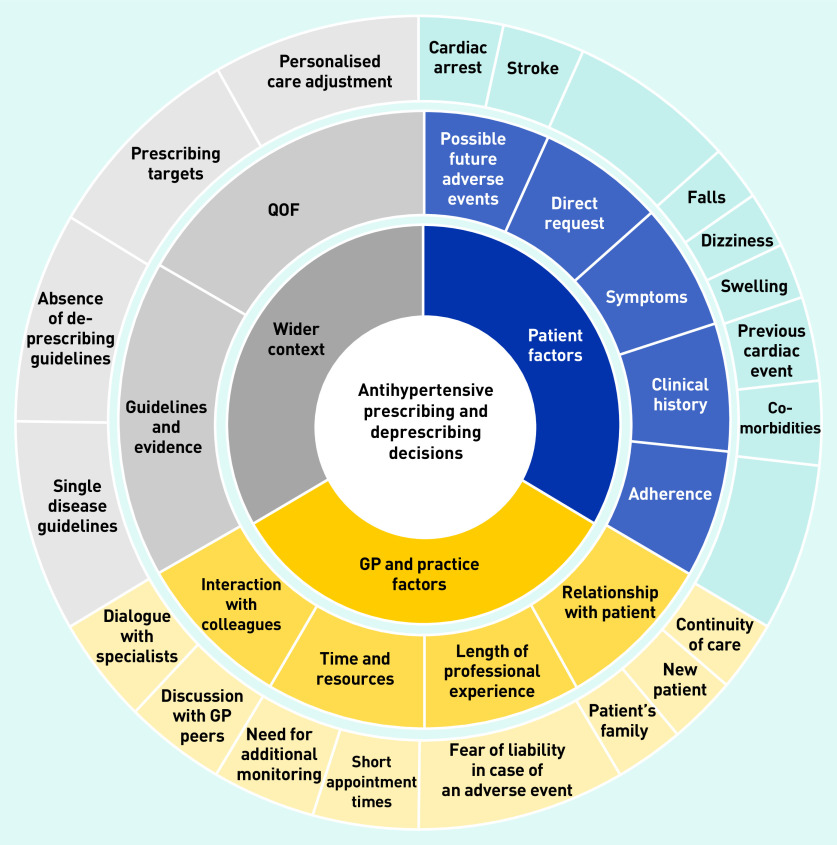
*Influences on antihypertensive prescribing and deprescribing decisions for primary care patients. QOF = Quality and Outcomes Framework.*

**Figure 2.**
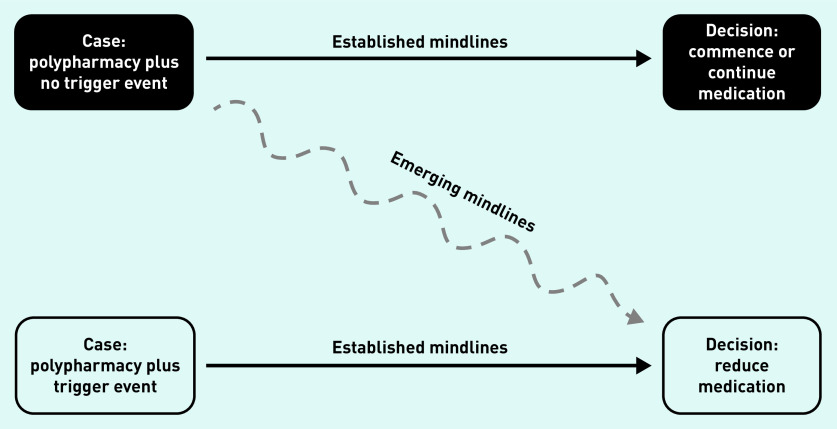
*Polypharmacy and medication reduction mindlines.*

Specifically, alongside the absence of evidence for deprescribing antihypertensives and the concurrent lack of guidelines in this area, this study’s analysis identified that GPs highlighted pressures to meet imposed prescribing targets; expectations of the patient’s amenability towards medication changes; likely concerns of and ability to communicate with the patient’s family and/or carers; and current resource constraints (for example, staff time required for additional monitoring). As a result, antihypertensive medication reduction was not common practice, usually only occurring reactively following specific trigger events, such as a recent fall. Here, decision pathways were clear for most GPs. When asked to consider proactive medication reduction to address polypharmacy in a patient, the most reasonable action was less clear. A greater readiness to consider medication reduction in these circumstances was apparent in GPs with more experience, and, consequently, more established mindlines developed over their years in practice. Influences on antihypertensive prescribing and deprescribing decisions, and the development of polypharmacy and medication reduction mindlines, are given in Figures 1 and 2. The complexity of these practices are discussed in more detail below.

### Established practices in medication decision-making

GPs outlined a number of scenarios in which decisions to reduce or remove medication — or to continue it — were often straightforward to make. In the case of deprescribing, GPs were more likely to consider, and were more confident about, withdrawing an antihypertensive drug if patients reported a side effect of such medication (for example, dizziness or swollen ankles) or if they were concerned about what they referred to as ‘pill burden’, or had a recent history of falls. Additionally, GPs were more likely to consider deprescribing antihypertensive medications with complicated side effect profiles, perceived procurement difficulties, or that were absent in updates of the NICE guidelines. However, antihypertensives were not necessarily the ‘go-to’ choice for deprescribing in cases of polypharmacy, where other medications might be withdrawn first:

*‘If people say: “I’m taking too many tablets”, then I would look at them and see which ones I could cut out. But the antihypertensives wouldn’t be at the top of that particular list, they would be the ones I would hang on to, and I would try and cut out other ones first. If someone is on a small dose of amitriptyline, because they have perhaps had a bit of neuropathic pain and the condition is minor, it has a side effect profile that’s anticholinergic, and for the elderly you try and reduce the cholinergic load. So I would be more inclined to stop something like that than to stop their lisinopril, for example*. *’*

(GP12, 12 years of experience)

In the case of reviewing medication regimens, absence of trigger events or adverse effects usually resulted in continued polypharmacy:

*‘We tend not to actively stop them unless* [patients] *have symptoms. If it ain’t broke, why fix it?’*

(GP08, 14 years of experience)

### Scarcity of explicit sources of knowledge and guidance in medication reduction decision-making

GPs attempting to consider deprescribing were aware of the increased interest in reducing polypharmacy, but also encountered a wide range of specific arguments against medication reduction. The majority of GPs interviewed said that the lack of guidelines was a major barrier to deprescribing antihypertensive medication in older patients who were not experiencing obvious side effects or other complications. An absence of guidelines on managing hypertension in the context of comorbidity, and the multiplicity of single-condition guidelines, added to the difficulty of making deprescribing decisions:

*‘I’d say the guidance is useful for starting antihypertensives,* [but] *there’s little guidance about withdrawing them and there’s never been any guidance as to timeframes and how quickly you should be up-titrating or downtitrating.’*

(GP04, 3 years of experience)

Prescribing decisions were made with an eye to requirements set out within the QOF.^48^ While GPs appreciated the option to make the right decision for the patient, facilitated by the possibility of making an exception report from the QOF for such patients where necessary (currently referred to as a ‘Personalised Care Adjustment’),^8^ they were also wary of the burden of additional regulatory inspections if targets were not met:

*‘We’re a bit reluctant to exempt because they think we’re cheating. And if you exempt too many people then you get an inspection and then we’re all sort of looking over our shoulder about this. So, that’s very difficult because the system, which is meant to be helping people, is actually harming people at that point.’*

(GP15, 6 months of experience)

This study found that GPs with more professional experience appeared more likely to deviate from national guidelines on acceptable blood pressure levels in patients aged ≥80 years and were better equipped to draw on their tacit knowledge about how to deprescribe. Less experienced GPs appeared more likely to continue polypharmacy in patients without new clinical symptoms, justifying their reluctance to deprescribe by the lack of explicit evidence of benefit on deprescribing:

*‘There are obviously targets to be met for people with diabetes in terms of blood pressure and things like that, but equally, I’m allowed to, where it’s clinically appropriate, exceptionalise people anyway, so I can think and make that decision*. *’*

(GP10, 10 years of experience)

*‘Is* [the patient] *going to have an increased risk of heart attacks or strokes from stopping* [antihypertensives] *, at this age group? Probably not, but can I be a hundred per cent certain that she isn’t going to go and have a heart attack or stroke? No.’*

(GP01, 1.5 years of experience)

### Patient considerations in medication reduction decision-making

Despite the scarcity of explicit guidance on deprescribing, some GPs were still willing to consider reducing medication regimens in people taking multiple medications, while acknowledging how complicated these decisions could be. Notably, such GPs tended to have more years in practice. In considering the rationale for medication reduction, GPs referred to the importance of a patient’s medical history and comorbidities, notably renal function, diabetes, and/or previous history of strokes or cardiac events:

*‘You’ve got to walk the tightrope between not upsetting somebody’s kidney function, but actually getting their blood pressure under control, and then not allowing the heart failure to get a hold, and then, if the heart failure gets a hold and you end up using diuretics, then you upset the diabetes, and they all interact.’*

(GP11, 26 years of experience)

Concurrently, a number of GPs mentioned that some patients, especially those with prior experiences of stroke or cardiac events, might experience anxiety at the prospect of stopping medication that lowers the risk of those events. Given that common understanding of having a chronic condition includes taking medication for the rest of one’s life,^37^^,^^49^ GPs with longer professional relationships with their patients might experience less resistance when suggesting medication withdrawal. However, this was not always the rule:

*‘I think sometimes people are absolutely delighted at the prospect of not having to take quite so many tablets and actually quite welcome it. But people who have had a stroke in the past are worried about the possibility of having a stroke if you stop the antihypertensive. So, I think the way round that is to actually talk to people properly about the risks and the benefits*. *’*

(GP11, 26 years of experience)

Conversely, a poorly established relationship with a patient made it more difficult to bring up the possibility of withdrawing medication, as GPs were unsure of the patient’s preferences and did not want to cause them any unnecessary anxiety. As a result, in the absence of new medication-related symptoms, polypharmacy was likely to be continued:

*‘* [The patient] *’s new to the surgery, I don’t know him, I don’t know how he feels really about the tablets given that I didn’t start them myself. It might be his choice that he would like to continue taking those tablets as he’s been taking them for a while and not had any problems.’*

(GP01, 1.5 years of experience)

GPs also expressed a concern that older patients frequently struggle with adhering to their existing medication regimens, which meant that any changes — including withdrawal of medication — might require considerations beyond the clinical effects of removing a single drug.

Additionally, GPs recognised that even if withdrawal of antihypertensives seemed fairly safe from a clinical point of view, the possibility of a catastrophic event could not be entirely eliminated given the combination of a patient’s age and level of multimorbidity. This means that even experienced GPs might hesitate before making deprescribing decisions:

*‘Something’s happened, and then if you say: “He’s had a fall and his blood pressure’s low, I think we need to reduce the medication”. That’s a lot easier than the more subtle quality of life, you know, potential risk of side-effect. And moving on, I guess your own worry as a GP is that if you do stop his anti-hypertensives, if he has a stroke 2 weeks later, whether it be incidental or not, is that your fault or not?’*

(GP10, 10 years of experience)

Some GPs mentioned that they found themselves in a difficult position when their patients received specialist advice that was in conflict with more holistic primary care goals. Resolving these conflicts can undermine the patient’s trust, especially if the specialist’s recommendations cannot be easily integrated with patient’s preferences or priorities, and the GP’s perceptions of these:

*‘To be quite blunt, I am getting tired of my elderly, frail patients being discharged* [from hospitals] *on long-term preventative medication that is potentially harmful. I think* [consultants] *need to be thinking about what they’re discharging on; sometimes these polypharmacy patients could have a fall and end up in hospital. I would appreciate those doctors to be reviewing the polypharmacy, to be assisting us in this. It can’t just come from general practice*. *’*

(GP15, 6 months of experience)

### Additional sources of concern in medication reduction decision-making

Complex considerations impacting decisions to deprescribe also involved factors beyond the health conditions of individual patients. For instance, almost all GPs interviewed mentioned that family and carers must be taken into account when considering medication changes for older patients. There was a general recognition that decisions about medical care stretch beyond the doctor–patient dyad, irrespective of the patient’s ability to make independent choices:

*‘If* [the patient’s] *family were a little bit more, sort of, accepting that age doesn’t mean that you’ve got to take lots of medication. And if they were less demanding on health services to expect that we continue to do things, and that if you stop something it doesn’t mean that you’re being a bad doctor, it means you’re just really trying to improve the quality of life index rather than pill pushing*. *’*

(GP02, 15 years of experience)

Many GPs pointed to additional time and resources necessary to monitor a safe withdrawal of antihypertensives that had to be weighed against the possibility of an adverse event, or even litigation. This included the potential for additional or longer appointments, home monitoring of blood pressure, and home visits for housebound patients. This was one of the main reasons for doctors being less keen on changing medication regimens in patients with no specific antihypertensiverelated side effects:

*‘Is there anything that’s stopping me*, *other than the problem that it could take more time, it could take more appointments to facilitate a safe withdrawal of tablets, in terms of following up with repeat home blood pressure monitoring, repeat appointments as to why we’re going to stop them as she’s been taking them for such a long time and been fine with them.’*

(GP01, 1.5 years of experience)

## DISCUSSION

### Summary

In older patients with multimorbidity, deprescribing decisions relating to antihypertensives are not straightforward. In everyday clinical practice, prescribing and deprescribing decisions demand GPs trade off a variety of factors, including explicit pressures of prescribing targets, tacit and practical knowledge on polypharmacy appropriateness for individual patients, patient preferences, and even concerns from families wanting more active medical care for their loved ones. In cases where there are clear trigger events for deprescribing (such as specific symptoms, a fall, or an explicit request from the patient), mindlines are well established. Outside of such triggers, this study shows that mindlines lead to a cautious approach to deprescribing by GPs, and a ‘watch-and-wait’ approach is prevalent. Consequently, even those GPs who would be keen to reduce polypharmacy in their older patients with multimorbidity may find it difficult to justify withdrawing antihypertensives without an explicit clinical reason, even in the context of strong relational continuity.

Deprescribing outside of a trigger event was considered more frequently by GPs whose mindlines were informed by many more years of clinical experience. Newly qualified GPs were less confident about stopping preventive medication in patients with complicated regimens who were not experiencing side effects or another active clinical concern. The climate in which GPs practice makes them acutely aware of the threat of litigation following a deprescribing decision that led to an adverse event, making them less willing to consider medication reduction. Although GPs’ anxiety about stopping antihypertensives is likely to decrease as they gain more professional experience, their mindlines may form more readily if specific guidelines on reducing polypharmacy were available. Such guidelines will not become available without first generating robust empirical data from trials and (to a lesser extent) observational studies.

### Strengths and limitations

While this study fits in with other research focusing on barriers and facilitators to deprescribing preventive medication,^13^^,^^15^^,^^16^^,^^29^^,^^30^^,^^33^^,^^34^^,^^36^^–^^41^ it focuses on gaps in GPs’ in-practice knowledge and practice related to deprescribing antihypertensives, drawing on thinking around mindlines. Within the interview study, the use of chart-stimulated recall can add to the specificity of interview data and facilitate deeper reflection in participants.^50^ GPs were asked to hypothesise about deprescribing decisions in cases of specific patients, which required real-time articulation of reasons about why deprescribing did not occur. The findings of the study may be limited by the small geographical area in England from which GPs were recruited and by the relatively low number of participants.

### Comparison with existing literature

Clinicians develop mindlines through everyday clinical practice and interaction. The robustness of these mindlines depends on available information in the form of the clinical evidence base (and guidelines) as well as professional experience and informal knowledge exchange between peers. Participating GPs in this study’s sample have demonstrated how their decision-making about deprescribing draws primarily on their own and their colleagues’ professional experience.^20^ Where the evidence base on the need to deprescribe was perceived to be clearer, such as in response to a trigger event, GPs’ mindlines were well developed. In other scenarios, however, mindlines tended to emerge more readily only among those physicians who have been practising longer.

Evidence supports the significance of mindlines in understanding how clinical decisions are made in the context of individual patients’ circumstances and broader structural pressures.^21^^,^^29^^,^^51^ A recent example of the nature and impact of mindlines comes from a study of management strategies for atopic eczema.^51^ Despite available evidence, such as NICE guidelines and local emollient guidelines, providing evidence-based treatment was challenging for GPs, who often perceived changes to recommended emollients as economically motivated. Instead, GPs’ mindlines drew on their own and their colleagues’ experience of managing eczema as well as online resources. Interestingly, practitioners who had personal experience of eczema were also the most likely to articulate a nuanced understanding of eczema management, including robust knowledge of available products to treat it.^51^

A growing interest in deprescribing in older patients with multimorbidity has been fuelled by mounting evidence on the potential harms of polypharmacy over the last two decades,^11^^–^^14^^,^^27^ as evidenced by the changes in the QOF.^8^ However, these trends have yet to translate into specific guidelines that would equip GPs for stopping preventive medication with the degree of confidence with which they prescribe it.^17^^,^^31^ In this context, GPs must (and do) rely on their own and their colleagues’ experience when making decisions to deprescribe. Consequently, the decision to continue polypharmacy in a patient who is not experiencing any side effects may often simply seem both risk-averse and wise, as exemplified in this study’s interviews with GPs.

### Implications for research and practice

There is a rising need to secure evidence on when and for whom deprescribing is most likely to be beneficial, as current practice heavily relies on GPs’ experience and intuition. These tacit approaches to making complex decisions are critical to developing confidence to attempt deprescribing and may be strengthened through reflective practice. As future doctors will be dealing with an increasingly aged cohort of patients with multimorbidity, deprescribing as a skill will need a place in the medical training curriculum. At the same time, it is important to recognise that, given the complexity of individual people taking multiple medications, professional guidelines and training are unlikely to be sufficient to address each patient’s needs and preferences in the light of their complex medication regimens and possible adverse effects. Doctors will develop and rely on mindlines when making complex decisions about reducing polypharmacy: surfacing the nature of such mindlines — as in this study — is one way to share practice more widely. As time goes on, an improved evidence base on the impact of deprescribing and guidelines on when and how to deprescribe are likely to support the strengthening of mindlines around deprescribing, even without trigger events.
